# Challenges of Transcultural Caring Among Health Workers in Mashhad-Iran: A Qualitative Study

**DOI:** 10.5539/gjhs.v8n7p203

**Published:** 2015-12-16

**Authors:** Rana Amiri, Abbas Heydari, Nahid Dehghan-Nayeri, Abou Ali Vedadhir, Hosein Kareshki

**Affiliations:** 1 School of Nursing and Midwifery, Mashhad University of Medical Sciences, Mashhad, Iran; 2 Evidence-Based Care Research Center, Department of Medical-Surgical Nursing, School of Nursing and Midwifery, Mashhad University of Medical Sciences, Mashhad, Iran; 3 School of Nursing and Midwifery, Nursing and Midwifery Care Research Center, Tehran University of Medical Sciences, Tehran, Iran; 4 Department of Anthropology, Faculty of Social Sciences, University of Tehran, Iran; 5 Honorary Research Associate (HRA), UCL Department of Science and Technology Studies (STS), University College London, Gower Street, London, WC1E 6BT, United Kingdom; 6 Educational Science and Psychology, Ferdowsi University of Mashhad, Mashhad, Iran

**Keywords:** health care staffs, immigrants, transcultural caring, cultural competence

## Abstract

**Background:**

One of the consequences of migration is cultural diversity in various communities. This has created challenges for healthcare systems.

**Objectives:**

The aim of this study is to explore the health care staffs’ experience of caring for Immigrants in Mashhad- Iran.

**Setting:**

This study is done in Tollab area (wherein most immigrants live) of Mashhad. Clinics and hospitals that immigrants had more referral were selected.

**Participants:**

Data were collected through in-depth interviews with medical and nursing staffs. 15 participants (7 Doctors and 8 Nurses) who worked in the more referred immigrants’ clinics and hospitals were entered to the study.

**Design:**

This is a qualitative study with content analysis approach. Sampling method was purposive. The accuracy and consistency of data were confirmed. Interviews were conducted until no new data were emerged. Data were analyzed by using latent qualitative content analysis.

**Results:**

The data analysis consisted of four main categories; (1) communication barrier, (2) irregular follow- up, (3) lack of trust, (4) cultural- personal trait.

**Conclusion:**

Result revealed that health workers are confronting with some trans- cultural issues in caring of immigrants. Some of these issues are related to immigration status and some related to cultural difference between health workers and immigrants. These issues indicate that there is transcultural care challenges in care of immigrants among health workers. Due to the fact that Iran is the context of various cultures, it is necessary to consider the transcultural care in medical staffs. The study indicates that training and development in the area of cultural competence is necessary.

## 1. Background

One of the consequences of migration is cultural diversity in different societies. The cultural diversity, often caused by immigrants, has created challenges for health care systems (Kirkham, 1998). Studies have shown that Medical personnel’s experience of caring for culturally diverse patients are challenging and frustrating. Cultural knowledge and understanding of medical staff about patients’ culture is a crucial factor in providing effective care (Ciofi, 2005). Erlen has recommended the health professionals must initially understand the patients’ culture, and set aside any prejudice that may affect their performance (Erlen, 1998). To offer the proper treatment, it is necessary to make the health care personnel familiar with transcultural caring (Vydelingum, 2006).

Transcultural identify as involving, encompassing, or combining component of more than one culture. According to Madeleine Leininger, the pioneer of transcultural nursing, transcultural nursing is a substantive area of study and practice that focuses on the comparative cultural values of caring, the beliefs and practices of individuals or groups of similar or different cultures. Transcultural nursing is an area of expertise in nursing that responds to the need for developing global perspective within nursing practice in a world of interdependent nations and people (Maler-Lorentz, 2008). However health staff face with many challenges and difficulties to provide treatment and care appropriate to the culture. One of the greatest challenges in transcultural communication is when medical personnel and clients speak different languages (Ciofi, 2003).

Iran is a country with large numbers of immigrants, wherein live at least two million foreign immigrants. 96% of these immigrants are Afghan (1,453, 513 people), 3% Iraqi (50.000 people) and 1% Pakistani (about 15-17 thousand people) (Statistical book year, 2011). Meantime, Mashhad, due to being coterminous with Afghanistan and Pakistan and being pilgrimage, has the maximum amount of immigrants (Statistical book year, 2011). The majorities of Immigrants who live in Iran are Shia and because of their religion have immigrated to Iran. There are two groups of immigrants in Iran; those who have legal residency and those who live illegally. These two groups are different regarding right of working, education and refer to health Centre. Immigrants who live legally have right to access to the health care, but they don’t have insurance and the cost of treatment is very high. They have right to work but they must do low-level jobs. Illegal Immigrants have not right to work and hospitals, especially public ones, do not accept and admit them. Most of immigrants cannot refer to hospitals because of the cost of treatment, so prevalence of chronic disease is high among them (Otoukesh et al., 2012; Koepke, 2011; Squire & Gerami, 1998; Olszewska, 1982).

On the other hand, recent studies have shown that immigrants give lower quality care than the resident population (Cioffi, 2005; Nelson, 2002; Dias, Severo, & Barros, 2008). Several studies have pointed out that immigrants have often less access to the health system than settlement (Adamson, Chaturvedi, & Donovan 2003; Dyhr, Andersen, & Engholm, 2007; Smaje, 1997; Hjern, Persson, & Rosen, 2001). Neglect, abuse and marginalization are parts of day-to-day experiences of the immigrants (Oddone, Wienberger, Freedman, & Kressin, 2002). Literature review showed that there are some qualitative studies about nurses and midwives’ experiences of caring immigrant and minority patients (Kirkham, 1998; Boi, 2000; Cioffi, 2004; Khanyile, 1999; McKinley, & Blackford, 2001; Murphy, & Clark, 1993; Dias Gama, Cargaleiro, & Martins, 2012; Zwane, & Poggenpoel, 2000). Studies on the health care staffs’ attitudes towards immigrants are almost quantitative and examined the health cares’ views through a questionnaire (Dias, Severo, & Barros, 2008; Michaelsen, Krasnik, Nielsen, Norredam, & Torres, 2004; Nielsen, Krasnik, Michaelsen, Norredam, & Torres, 2008). There are few qualitative studies on medical personnel’s experience and views to immigrants (Abbott, & Riga, 2007; Priebe et al., 2011). Considering to different cultural background and large number of immigrants in Iran, it is essential to pay attention to transcultural caring in this society. Also, no study has been done on this issue in Iran; therefore, in this study we aimed to examine the medical personnel’s experiences, doctors and nurses, of caring from immigrant patients.

### 1.1 Objective

This qualitative study describes the medical staffs’ experiences of caring for immigrants.

## 2. Methods

This is a qualitative study, since experiences are subjective; and only through qualitative study we can discover real feeling and attitudes of people in that context. In terms of qualitative research, the researchers have used content analysis approach. This study has been done in Mashhad-Iran in 2013. Mashhad was chosen as place of study, since many immigrants live there due to its coterminous with Afghanistan and Pakistan and being pilgrimage. The most referred immigrants’ hospitals such as Hasheminejad, Imam Hossein and Imam Hadi were selected. Fifteen health workers who met the inclusion criteria: work in the most refer immigrants’ hospital and clinic, having a bachelor degree or higher, five years or more experience working with immigrant in that clinics or hospitals and volunteered to join, entered to the study. Exclusion criteria were unintended to participation and having work experience less than 5 years. To understand the factors influencing the experience of medical personnel, purposive sampling was used.

In this study, the purpose of study was initially explained to the personnel and if they were willing or qualified, a written consent would be obtained from them. Then, the time and place of interview was determined according to the participants’ will. To collect data, semi-structured interview was used. Participants were free to express their feelings and experiences. First, an open question was asked of participants like “what is your experience of caring from immigrant patients”. The researcher without directing the question, tried to obtain their experiences through in-depth dialogue.

Each session may last for 45 minutes to 1 hour in a comfortable and quiet place. Interview was recorded with a digital recorder. In addition to recording, face and body gestures (body language), pauses and non-verbal gestures was noted by the researcher.

In qualitative research, the participants’ selection should be determined based on data needs. Thus, a guiding principle for choosing participants is data saturation. In this study, interviews with health staffs have continued until the data saturation was obtained. Sample selection process has continued until no new data was appeared during data collection.

Ethical consideration: To keep ethical considerations, the ethics committee of Mashhad University of Medical Sciences has approved the study. The researchers explained the goal of study to the participants; although their right to refuse participation at any time during the study was also stressed. In addition, the patients who consented to participate in the study were asked to sign a consent form. Permission to use an audio tape recorder during the interview sessions was obtained from health workers.

Data analysis: Data analysis was performed simultaneously with data collection. Data were entered to Max QDA software. For data analysis, the latent qualitative content analysis by Graneheim and Lundman was used. Data were classified and united. Uniting data includes raw coding and giving units meaning and classification includes grouping units of meaning or logic to large classes and the main category based on the similarity (Graneheim & Lundman, 2004). To increase reliability and validity of data, different methods such as allocating sufficient time, in-depth interviews with participants, explaining the objectives of the study and returning codes to participants to verify their accuracy were used.

## 3. Result

Fifteen participants (seven doctors and eight nurses) entered to the study. Among doctors, two were female and five male and had experience ranging from 5 to 20 years. Five nurses were female and three were male and they had experience ranging from 5 to 15 (see in Table 1). Each interview lasted approximately 45 to 60 minutes. The results were in the main named as challenge in transcultural caring and were presented at four categories of “communication barriers,” “irregular follow up,” “lack trust” and “cultural - personal trait”. The main core, categories, and subcategories are presented in Table 2. Each category has been discussed in some detail.

**Table 1:** Participant’s characteristics

Participants /Field		Level of education	Experience (year)	Age (year)	Gender	Place of work
P1	Doctor	GP	8	45	Female	Sakhteman- clinic
P2	Doctor	Ophthalmologist	15	55	Male	Khatamol-anbiya-hospital
P3	Doctor	Gynaecologist	10	43	Female	Golshar – clinic
P4	Doctor	GP	12	54	Male	Golshar-clinic
P5	Doctor	Internal Medicine	5	38	Male	Bolvar Tabarsi-office
P6	Doctor	GP	20	57	Male	Golshar-office
P7	Doctor	Internal Medicine	7	43	Male	Tabarsi-Office
P8	Nurse	Bachelor	5	32	Female	Sakhteman-clinic
P9	Nurse	Bachelor	6	35	Female	Sakhteman-clinic
P10	Nurse	Bachelor	13	40	Male	Bolvar Tabarsi-clinic
P11	Nurse	Bachelor	10	38	Male	Golshar-clinic
P12	Nurse	Bachelor	5	31	Female	Bolvar Tabarsi-clinic
P13	Nurse	Master	10	37	Male	Imam Hadi
P14	Nurse	Bachelor	15	38	Female	Hasheminejad- paediatric ward
P15	Nurse	Bachelor	6	29	Female	Imam Hossein –OB

**Table 2:** Themes and subthemes

Theme	Subtheme
**Communication Barriers**	Different languages
	Different Interpretation of signs and symptoms
**Irregular Follow- up**	Residential financial problem
	Inadequate information
	More drug, more qualified doctor
**Lack of trust**	Frequent changing doctor
	Disregarding staff recommendation
**Cultural- personal trait**	Low communication skill
	Men’s decision role in the treatment of women
	Being shy

### 3.1 Communication Barriers

Participants raised communication barriers in two different types. The first type includes language barriers due to different languages or dialects. Most personnel were not able to understand what immigrants said and vice versa. As indicated by GP:

“What was the main problem in dealing with immigrants? When I first started my job here, I had lots of problem with the language of immigrants. Some of them even couldn’t understand me. It was a great challenge because I had to give some information to my patients, but it was interrupted in this way and I was not happy (general practitioner, participants 1)”.

The second type of communication problems includes different interpretation of signs and symptoms of the disease between staff and patients. Immigrants were confusing personnel with different expression of signs and symptoms. Health workers indicate this problem as follows:

“A patient referred to us several times and asked us that he feels his back is loose and need some information for this problem. We did not understand what he meant. He didn’t explain more, as he was embarrassed. After a few sessions, we finally realized that he means his libido was low (nurse, participant 7).

“I often can understand their language because it is Persian, but I have a little difficulty in describing some of their sign and symptom. For example, they say I get hot hot and it is difficult unless the doctor understands what they culturally mean (general practitioner, participant 4)”.

### 3.2 Irregular Follow up

Personnel pointed that immigrants have not regular follow-up; therefore the prevalence of chronic disease is common among them. Health workers indicated to two reasons for irregular follow up; consisting of residential- financial problems and inadequate information.

The first subcategory is residential- financial problems. Most immigrants live in tragic situations in poverty and they could not afford cost of treatment. Cost of treatment in Iran is very expensive for immigrant, because most of them have not insurance. These financial problems in immigrants have created some challenges for disease follow up.

One of the doctors explained this reality:

“Immigrants who living in this area and we deal with them are those who are in short financially. Most of these immigrants do self-treatment and they would refer for treatment if they get worse. That’s why chronic diseases are prevalent among them. I had a couple of cases referred to me and they need more Para-clinical examination and follow-up, but they cannot, which is mostly due to financial issues (Internal medicine, participant 5)”.

Some doctors and nurses face challenge due to the lack of residential permit in immigrants. Undocumented immigrants don’t have right to admit in the public hospitals; even if they are in an emergency situation. These situations create some problems for doctors, because they are not able to refer for further treatment. One of doctors indicated it such as:

“A patient referred to me with glaucoma. I prescribed him to do emergency surgery in… hospital as soon as possible. When he referred to the hospital, they didn’t admit him because of illegal resident. He came back to my office and asked help me. I admitted him in the hospital on my own responsibility, but it must be legally bad for me. We don’t know what to do in this situation; we have to do something because we have a commitment (ophthalmologist, participant 2)”.

The second subcategory is inadequate information. One of the reasons for late referring to the doctor in migrants was lack of information about health and health seeking. Because of inadequate information, they do not worry about their illness and are careless about themselves. The GP is pointed to this reality:

What is interesting about immigrants is that they are careless regarding health. One of my patients has a blood pressure above 20, but it does not matter too much for him. I told him your pressure is very high, you may have a heart attack, it is dangerous, but it is not so important for him, He is so carefree. (GP, participants 6).

Participants stated that disease could be diagnosed incidentally in some immigrants. It means that they ignore or tolerate the problem until it is accidently diagnosed. The GP said that:

“I may see here very malignant hypertension, BP 27, the patient may have a high blood pressure for years or may come for another problem. For example, a patient came to the clinic a couple of weeks ago with a blood pressure 26.5; I do not remember its diastole well. When I checked his blood pressure I even doubt, and checked it again. Then I wanted another barometer, but the result was the same. The patient was immigrant that he came for another problem, he couldn’t believe as well. They don’t refer for their health. I had a patient that was first diagnosed with diabetes with blood sugar more than 300 (GP, participant 4).

### 3.3 Lack of Trust

Another challenge among personnel is mistrust of immigrant toward them. Immigrants show this mistrust in form of some beliefs and behaviors such as; more drugs more qualified doctors, frequent changing doctor and disregarding staff recommendations.

Some immigrants believe that the doctor, who prescribed more medication, has better diagnosed their disease. Next belief, which is common among immigrants, especially in those with low education, is belief to the injections. They think that injection is more useful than other drugs for diseases, and when they refer for treatment, they just ask for injections. As explained by one of our participant:

“One of my concerns regard to immigrants is requesting injectable drugs. They believe that their disease is treated only with injections. It’s highly prevalent among immigrants, that Afghans called it Pichkari. When they come here, they asked me Pichkari, if I don’t prescribe injection they leave office unsatisfied (general practitioner, participants 1)”.

According to the staff’s statement some immigrants accept education hardly and often resist on training and staff talks. They are convinced very hard, because they do not trust health workers. Most personnel complain about the fact that immigrants don’t pay attention to the recommendations. A nurse indicated it:

Some immigrants refer to me for blood pressure control. They told me their blood pressure is high. When I check their pressure I see its ok. But they don’t accept it and they ask to retake. I retake and ensure them that they are well, but they don’t accept me (nurse, participant 11).

Most medical personnel stressed that migrants pay more attention to neighbors’ advices than them, and this is one of the major problems to seek treatment. Participants declared that immigrants are often distrustful, they do not trust the staff, so they do not accept the teachings.

One of common behavior among immigrants is that they constantly change their doctor. This behavior also emerged from mistrust to physician. In fact, it is believed that if the doctor is good, they should feel better with one or two doses of drug and if they don’t, they conclude that their illness is not diagnosed properly and they change the doctor. They refer several doctors for a disease and each time they don’t complete the treatment.

One of doctors pointed out to this subject:

“One of concern regarding immigrants is following up the diseases. Patients come to me and receive some drugs and not completing them, they refer to another doctor and then another and another and a small problem will be converted to a complicated issue (internal medicine, participant 5)”.

### 3.4 Cultural-Personal Trait

Some cultural - personal trait of immigrants were considered as challenge to treat and care them. These cultural - personal trait are including:

- Being shy: One of the barriers to treatment is feeling shy that exists often in genital - urinary diseases or rectal problems. This behavior is more common in women, but men also have this problem and shy to expressing their disease. Also, due to the religious beliefs, they often prefer to be examined by a same sex doctor. Some women, especially elderly, prevent from gynecological examination. One of our participants pointed out to this issue:

“Once a woman about 60- 70 years referred to me. She just complained of irritation. When I told her go for internal examination, she blamed me that shame on you, you are as old as my daughter, I had 9 pregnancy and childbirth and no one have seen me. I insisted more and she went on bed for gynecological examination. I realized she had complete prolapse of the uterus that half of the uterus was out of vagina. I asked her if she felt something out of her, she said yes I did. I asked why you didn’t say it. She told I shied to tell my daughter and granddaughters (Gynecologists, participant 3)”.

- Men’s decision role in the treatment of women: Patriarchal in culture of immigrants has caused the major role of decision-making in the family to be the responsibility of men. Participants stressed that in some families, the authority is to the extent that women cannot follow up their disease if their husband was unsatisfied. Of course, especially if the disease is related to the uterus and reproductive system, the men decision-makers are more pronounced. As indicated by a doctor:

I had a patient who had polyp in uterus. I gave her some common medications to stop bleeding and advised her for emergency surgery. After a while, she came back and said: “my husband did not let me for surgery, he told me if I operates my uterine he would divorce me or remarry (Gynecologists, participant 3).

- Low communication skill: Some immigrants don’t have good communication with others. They often isolated and are not sociable. Staff stated that immigrants often cannot communicate and we have to pin them down. They are often convinced, shy and unexpected. Inabilities to communication harm their health and prevent health workers to easily understand their problems.

## 4. Discussion

This study has been done aiming to explore the medical personnel’s experience of caring for immigrants and lead to the formation of four categories: communication barrier, irregular follow up, lack of trust and personal – cultural traits. It was indicated that there are transcultural challenges regarding the care of immigrants among health care workers. Due to the large number of immigrants and different cultures in Iran, paying attention to this subject is necessary. So this study provides unique and innovative result for improving quality of health care in Iran.

Regarding the first category - communication barrier- many studies stated that one of the biggest problem in proper care for people with diverse culture and language is communication barrier (Scheppers, Dekker, Geertzen, & Dekke, 2006). Several possible reasons have been proposed for communication barrier, including different interpretations of signs by immigrants and health workers, different language in two groups and different health needs (Adamson, Chaturvedi, & Donovan, 2003). Furthermore, our study revealed that there are two kinds of language barrier; firstly different language and secondly different interpretations of the signs and symptoms of disease. On the other hand according to Steeger and Lipson, the effective trans-cultural communication includes emotional, cognitive and behavioral strategies. Emotional strategy includes respect, appreciate and feel comfortable with the cultural differences, learning through cultural exchange, ability to have unprejudiced behavior and awareness of cultural values. Cognitive strategy involves acquiring knowledge about different cultures and ability to understand the culture. Behavioral strategies include flexibility in verbal and non-verbal communication, ability to speak calmly and without accent. These strategies show that language is only small part of the communication process and cultural resources and realities of the patients should also be considered (31). Therefore, in our study the problem is low communication skills and familiarity with the transcultural care among health care workers. Unfortunately there is no proper training for medical student and staff in Iran for transcultural communication and cultural competency. In this regard, it is recommended to provide proper transcultural communication training for health care workers, to enable them to communicate effectively despite the language and culture difference.

Regarding to irregular follow up, health care workers pointed out that immigrant have not suitable follow up because of financial-residential problem and low information. Different studies pointed out the residential- financial problems of immigrants as barrier for health care seeking (Scheppers, Dekker, Geertzen, & Dekke, 2006). On the other hand, low knowledge and information about disease among immigrants is one of the problems that have indicated by different studies (Lipson & Omidian, 1997; Refuges watch, 2000). Lack of trust was another category in our study, which was confirmed by other research. Studies have indicated that mistrust toward health care system is more common among immigrants because of their situation and condition. They cannot communicate with health care properly, cannot understand their language and always feel inferior, which result in mistrust. In addition other studies in Iran showed that patients had lack of trust to personnel because of unequal and superior-inferior relation between the staff and patients (Vaseli, Dehghan-Nayeri, Borim-Nezhad, & Vedadhir, 2015). Ignoring and not meeting the emotional and psychological needs of the patient was evidence of non-interactional communication in Iran. This unfair relation resulted in patient’ distrust toward the staff.

Regarding immigrants’ cultural – personal traits in our study, participants stated to the special cultural features that could limit their treatment and care. Individual cultural behaviors are varies from person to person and society to society. By recognizing these cultural behaviors, substantial steps can be taken to resolve the defects. Meyer and colleagues provided four major challenges for cultural competency in health caregivers. First, there is a different understanding of disease in different areas; for example, some patients according to their culture do not want to talk about sexual problems. If we respect culture, won’t we ignore the sexual problems? (Meyer, 2012). In our study, there was also this challenge for health care staff. Patients disagree with discussing or examining the sexual issues and even prevent from doing so. Here, a challenge is created for the staff whether they should respect their cultural restrictions or do the treatment. Men power in decision-making for women’s health care had created problems for staff, because entering the family environment was considered as annoying action. On the other hand, if no intervention has been done, the person may die due to the disease.

The second and most important challenge is communication and mastering different languages, that in our study, the challenge existed somehow. The next challenge is race, if a group has a higher education level, we should respect them or due to cultures, we must try avoiding the culture of dominating and the fourth is trust which is obtained hardly because of different cultures (Meyer, 2012). In our study, most personnel have indicated that immigrant mistrust health care workers and this caused they not pay attention to their recommendation.

## 5. Conclusion

In conclusion we recommend considering the transcultural caring and cultural competency among medical staff due to the fact that Iran is a ground for different cultures and also due to the pilgrimage of Mashhad and having large numbers of pilgrims annually from neighboring countries. Considering domestic and foreign cultures can improve the quality of healthcare services provided.

This study has implication for research and practice. Despite the large number of immigrants and various cultures in Iran, it is the first study that works on transcultural caring. It is very important subject in health area that should be considered by scholars and further research about this topic is necessary. Furthermore, for practice it has some implication; pay attention to needs of immigrants and especially their cost of treatment should be considered by UN, also holding some training course to enhance health information of immigrants is recommended. As well as holding transcultural caring course is needed for health care workers.

This study has some limitations: One of our limitations is that personnel was very busy and had no time for interview. They didn’t like to interview beyond their job and in job environment (hospital, clinics or office), they were very busy. So we asked 10 nurse and 10 doctors to participate, but some of them did not accept our request. Next limitation is that in our study we consider health workers as nurse and doctors and did not consider other groups of health workers. Finally, in addition to interview, using other data gathering strategies such as observation helped strengthening the data.
